# 3D-printed PCL framework assembling ECM-inspired multi-layer mineralized GO-Col-HAp microscaffold for in situ mandibular bone regeneration

**DOI:** 10.1186/s12967-024-05020-1

**Published:** 2024-03-01

**Authors:** Yanqing Yang, Huan He, Fang Miao, Mingwei Yu, Xixi Wu, Yuanhang Liu, Jie Fu, Junwei Chen, Liya Ma, Xiangru Chen, Ximing Peng, Zhen You, Chuchao Zhou

**Affiliations:** 1 Department of Plastic Surgery, Tongren Hospital of Wuhan University (Wuhan Third Hospital), Wuhan, 430060 China; 2 Division of Biliary Surgery, Department of General Surgery, West China Hospital, Sichuan University, Chengdu, 610041 China; 3 Department of Plastic Surgery, Beijing Hospital of Integrated Traditional Chinese and Western Medicine, Beijing, 100038 China; 4 Department of Dermatology, Renmin Hospital of Wuhan University, Wuhan, 430060 China; 5 The Centre of Analysis and Measurement of Wuhan University, Wuhan University, Wuhan, 430072 People’s Republic of China

**Keywords:** 3D print, ECM-inspired, Graphene oxide, Biomimetic mineralization, Bone regeneration

## Abstract

**Background:**

In recent years, natural bone extracellular matrix (ECM)-inspired materials have found widespread application as scaffolds for bone tissue engineering. However, the challenge of creating scaffolds that mimic natural bone ECM’s mechanical strength and hierarchical nano-micro-macro structures remains. The purposes of this study were to introduce an innovative bone ECM-inspired scaffold that integrates a 3D-printed framework with hydroxyapatite (HAp) mineralized graphene oxide-collagen (GO-Col) microscaffolds and find its application in the repair of mandibular bone defects.

**Methods:**

Initially, a 3D-printed polycaprolactone (PCL) scaffold was designed with cubic disks and square pores to mimic the macrostructure of bone ECM. Subsequently, we developed multi-layer mineralized GO-Col-HAp microscaffolds (MLM GCH) to simulate natural bone ECM's nano- and microstructural features. Systematic in vitro and in vivo experiments were introduced to evaluate the ECM-inspired structure of the scaffold and to explore its effect on cell proliferation and its ability to repair rat bone defects.

**Results:**

The resultant MLM GCH/PCL composite scaffolds exhibited robust mechanical strength and ample assembly space. Moreover, the ECM-inspired MLM GCH microscaffolds displayed favorable attributes such as water absorption and retention and demonstrated promising cell adsorption, proliferation, and osteogenic differentiation in vitro. The MLM GCH/PCL composite scaffolds exhibited successful bone regeneration within mandibular bone defects in vivo.

**Conclusions:**

This study presents a well-conceived strategy for fabricating ECM-inspired scaffolds by integrating 3D-printed PCL frameworks with multilayer mineralized porous microscaffolds, enhancing cell proliferation, osteogenic differentiation, and bone regeneration. This construction approach holds the potential for extension to various other biomaterial types.

**Graphical Abstract:**

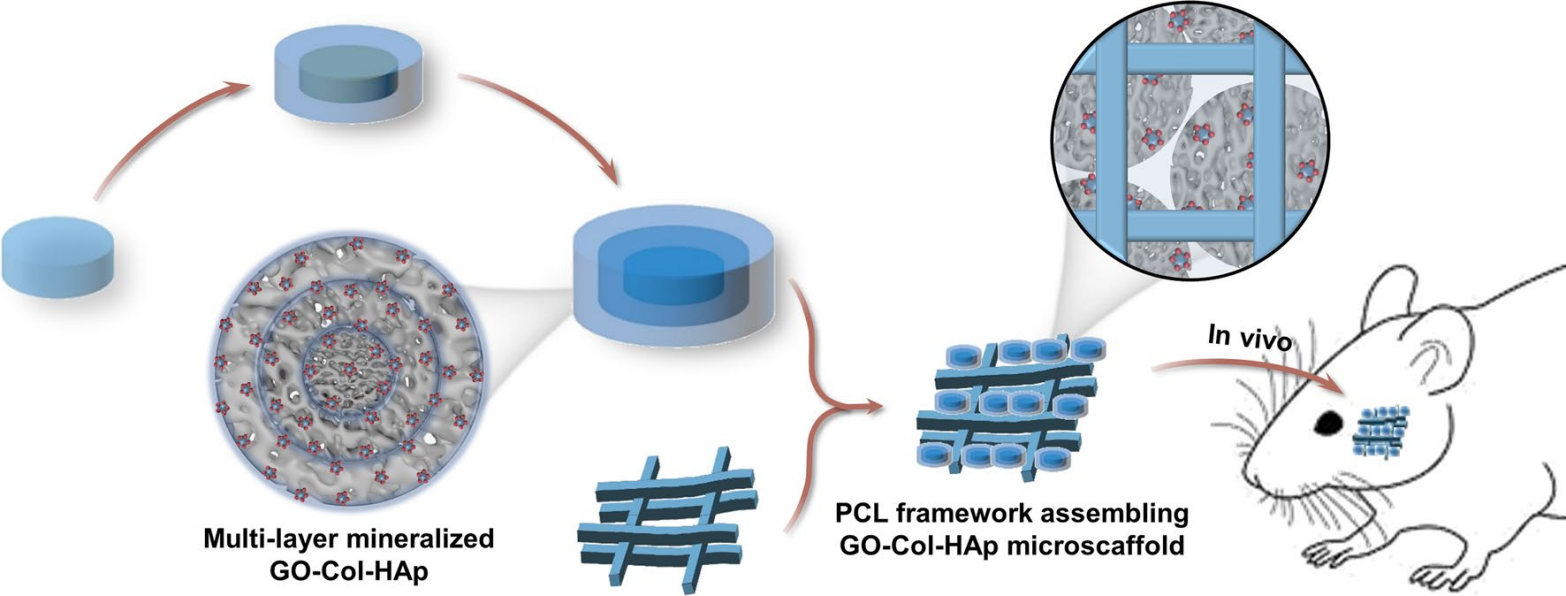

**Supplementary Information:**

The online version contains supplementary material available at 10.1186/s12967-024-05020-1.

## Introduction

Craniomaxillofacial bone defects in dentistry are frequently observed and arise from various causes, including trauma, tumor growth, bone infection, or hereditary malformation [1]. The repair and regeneration of these defects pose significant challenges due to the intricate nature of the structures involved and the complex biomechanical and physiological environment [2]. Presently, approaches employed for the reconstruction of craniomaxillofacial bone defects encompass the use of autografts and allografts, rigid fixation techniques, and free tissue transfer [3]. Nevertheless, these strategies are accompanied by several limitations, including nonunion, donor site morbidity, ethical concerns, potential immunogenic rejection, and supply constraints [4]. Therefore, there is a pressing need to develop bone grafts that mimic bone tissue structure and function, exhibiting favorable biocompatibility, osteoconductivity, and mechanical stability.

In recent years, 3D printing technology has gained prominence in the construction of bone repair grafts characterized by intricate architectures and impressive mechanical properties [5–7]. Utilizing techniques like selective laser sintering (SLS), stereolithography (SLA), 3D bioprinting (BP), and fused deposition modeling (FDM), these grafts demonstrate robust mechanical attributes and desired structure [8]. Moreover, the application of advanced 3D-printed materials, such as titanium, hydroxyapatite (HAp), β-tricalcium phosphate, poly (lactic-glycolic) acid (PLGA), and polycaprolactone (PCL), further enhances biocompatibility and mechanical properties [3, 9, 10]. Among the various materials under consideration, PCL exhibits significant advantages in the fabrication of bone scaffolds using 3D printing technology. PCL has gained widespread adoption owing to its favorable biocompatibility and convenient processing characteristics, as evidenced by its approval by the United States Food and Drug Administration (FDA). Furthermore, the composition and structure of PCL can be readily modified to suit specific requirements [10, 11]. Nevertheless, the development of these scaffolds has been hindered by their inability to replicate the distinct microenvironmental cues of the natural bone extracellular matrix (ECM) [12].

The freeze-drying technique is a cheap, efficient, simple, and environmentally friendly approach to developing porous and interconnected as well as a high surface area that mimics the natural structure of ECM [13–15]. Moreover, type I collagen (Col) is a prevalent ECM component, notably in bone, skin, and tendon tissues [16]. Hybrid scaffolds comprising freeze-dried collagen have gained attention as bone grafts, exhibiting excellent biocompatibility, facile customization, and ECM-like properties [17, 18]. Nonetheless, the osteoconductivity of collagen-based scaffolds remains an area of improvement [19].

In the context of bone development, type I collagen serves as a foundation for hydroxyapatite mineralization, a principal component of natural bone [20, 21]. Drawing inspiration from this mineralization process, a promising technique involves coating scaffold surfaces with HAp through simulated body fluids (SBFs), which emulate blood plasma ion concentrations [22, 23]. This biomimetic HAp-coating approach has been successfully applied to diverse materials, including metals, ceramics, and polymers, enhancing biocompatibility and osteoconductivity [24, 25]. However, within 3D porous scaffolds, the biomimetic coating exhibits limited ability to uniformly deposit HAp throughout their inner regions, leading to uneven distribution and inadequate thickness [26].

Over the years, significant efforts have been undertaken to overcome the limitations of HAp coating in SBF. For instance, Liu X et al. found that incorporating graphene oxide (GO) into cellulose acetate nanofibrous scaffolds increased biomimetic mineralization efficacy by providing additional nucleation sites for HAp deposition [27]. A. L. Oliveira et al. demonstrated that dynamic conditions facilitated thicker apatite layers and higher mineralization efficiency compared to static conditions [28]. Altering the 3D scaffold structure also contributes to achieving uniform HAp distribution; Zhou C et al. introduced a pearl-inspired microgel with multi-layer mineralization to achieve comprehensive mineralization [29].

**Fig. 1:**
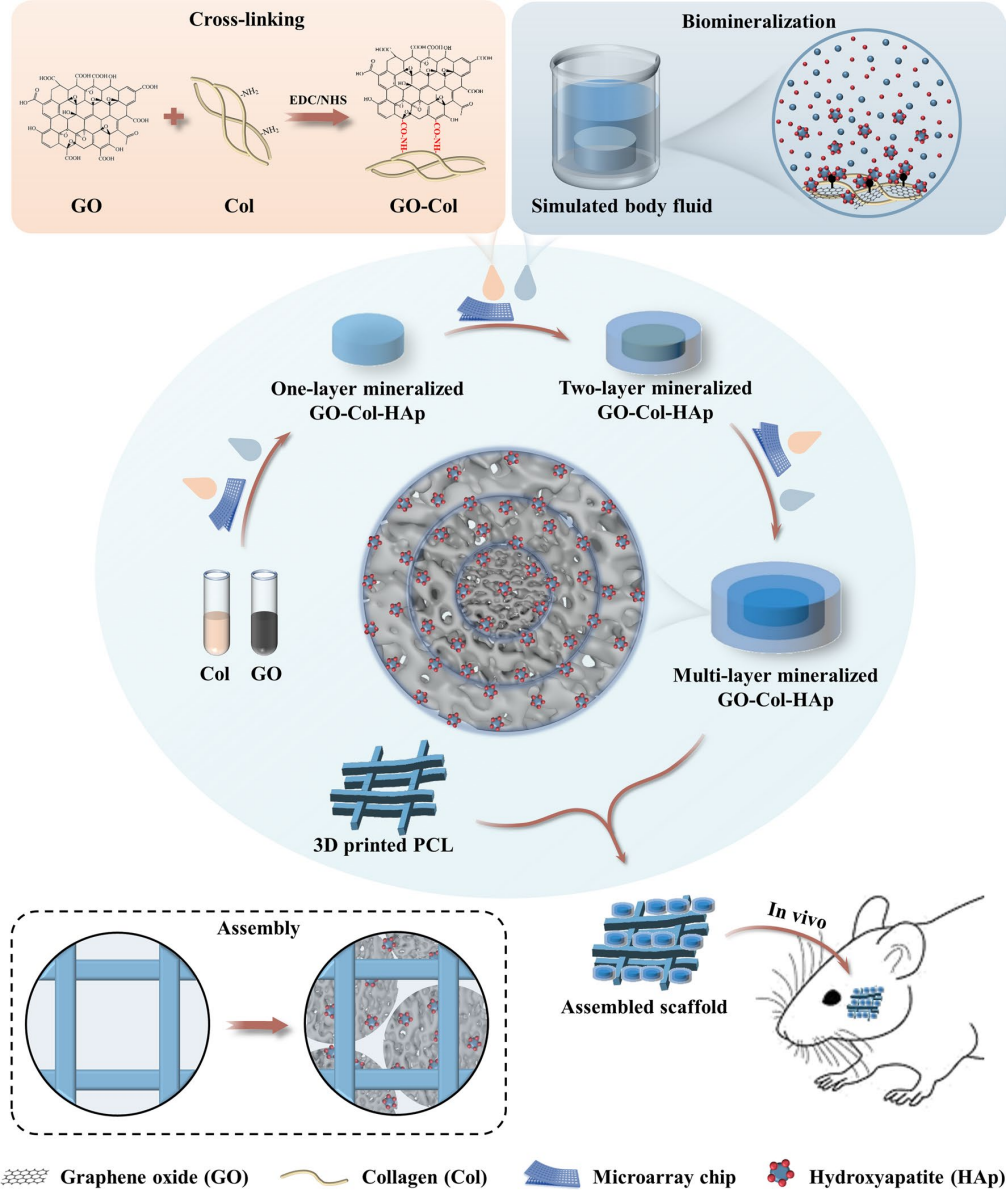
Schematic illustration of the fabrication of 3D-printed polycaprolactone framework assembling ECM-inspired multi-layer mineralized GO-Col-HAp microscaffolds and its application in mandibular bone regeneration

In line with these findings, we created multi-layer mineralized and GO-infused Col microscaffolds through freeze-drying to replicate the ECM environment (Fig. 1). These microscaffolds were subsequently incorporated into 3D-printed PCL scaffolds to provide appropriate mechanical support. Moreover, the multi-layer mineralized structure was harnessed to achieve uniform HAp distribution. The assembly of ECM-inspired microscaffolds and 3D printed PCL framework was designed to provide an ideal shelter for the proliferation and osteogenic differentiation of the host-derived cells. This study includes comprehensive in vitro experiments to assess ECM-like conditions, uniform HAp distribution, and the impact of assembled scaffolds on cell viability. Additionally, in vivo experiments were conducted to assess the bone regeneration capability within a rat mandibular defect model. Importantly, our study sheds light on a structural approach to achieve uniformly distributed HAp and the development of ECM-inspired scaffolds for effective craniomaxillofacial bone defect repair. Innovatively, the integration of 3D-printed PCL frameworks and ECM-inspired microscaffolds has potential applications in the field of biomaterial assembly.

## Materials and methods

### Materials and animals

PCL (*M*_*w*_ = 79–90 kDa, melting point 60 ℃) was purchased from Sigma-Aldrich, America. Graphene (500 meshes) was purchased from Acros Organic Company. Collagen was obtained from Chengdu Kele Biotechnology Co., Ltd, China. N-hydroxysuccinimide (NHS) and N-(3-Dimethylaminopropyl)-N-ethyl carbodiimide hydrochloride crystalline (EDC) were purchased from Sigma-Aldrich, China. Kokubo’s method was applied to prepare the simulated body fluid (SBF) solution [24]. Reagents associated with the preparation of SBF including acetic acid, NaCl, KCl, CaCl_2_·2H_2_O, MgCl_2_·6H_2_O, HCl, Na_2_SO_4_·10H_2_O, K_2_HPO_4_·3H_2_O, NaHCO_3_, and NH_2_C(CH_2_OH)_3_ were obtained from Sinopharm Chemical Reagent Co., Ltd, China.

Fluorescein diacetate (FDA), propidium iodide (PI), dimethyl sulfoxide (DMSO), and 3-[4,5-dimehyl-2-thiazolyl]-2,5-diphenyl-2-H-tetrazolium bromide (MTT) were purchased from Sigma-Aldrich, China. Fetal bovine serum (FBS), Low-glucose Dulbecco’s modified Eagle medium (L-DMEM), penicillin, and streptomycin were obtained from Hyclone, America. Phosphate buffered saline (PBS), and Trypsin–EDTA were obtained from Sinopharm Chemical Reagent Co., Ltd, China.

Male adult Sprague Dawley rats (4–6 weeks old, 180-220 *g* weight) were supplied by the Department of Experimental Animals, Tongji Medical College, Huazhong University of Science and Technology. All animal-related procedures were approved by the Animal Research Committee of the Tongji Medical College, Huazhong University of Science and Technology. All animals were sacrificed in accordance with the Standing Committee on Ethics in China (State Scientific and Technological Commission of China) after the experiments.

### Fabrication of the microscaffolds and the assembled PCL constructs

#### Fabrication of the microscaffolds

Graphene oxide (GO) was synthesized according to the modified Hummer method [30]. Three kinds of microarray chips (261 circle wells with 800 μmφ, 1200 μmφ, and 1500 μmφ diameters) were prepared by using polymethyl methacrylate (PMMA) assisted by laser prototyping technique [31]. GO solution (0.2% w/v in 0.1 mol/L HAc) and Col solution (4% w/v in 0.1 mol/L HAc) were mixed in equal volumes and sonicated for 30 min to obtain the homogeneous GO/Col solution.

The multi-layer mineralized GO-Col-HAp (MLM GCH) microscaffolds were synthesized by multiple encapsulation and biomineralization of the three kinds of microscaffolds (800 μmφ, 1200 μmφ, and 1500 μmφ). To fabricate the 800 μmφ GCH microscaffolds, the obtained GO/Col solution was transferred to a 800 μmφ microarray chip and subsequently frozen at – 20 ℃ overnight and freeze-dried at − 50 ℃ for 12 h. The fabricated 800 μmφ GO-Col microscaffolds were removed from the chip and cross-linked by EDC/NHS ethanol solution (EDC: NHS = 5:2, H_2_O:ethanol = 5:95) for 12 h, thoroughly washed in ddH_2_O, followed by immersing into SBF for 7 day, washed again with ddH_2_O, and finally obtained the 800 μmφ GCH microscaffolds (Additional file 1: Video 1). Next, to fabricate the 800–1200 μmφ GCH microscaffolds, the GO/Col solution was transferred to a 1200 μmφ microarray chip instead. Then, the 800 μmφ GCH microscaffolds were put into each well and subsequently freeze-dried for 12 h. Followed by removing the microscaffolds from the microarray chip, cross-linking in EDC/NHS ethanol solution, biomimetic mineralization in SBF for 7 day, and finally obtaining the 800–1200 μmφ GCH microscaffolds. In the end, to fabricate the MLM GCH (Three layers, 800-1200-1500 μmφ) microscaffolds, the fabricated 800–1200 μmφ GCH microscaffolds were put into 1500 μmφ microarray chip filled with GO/Col solution. After lyophilization, cross-linking, biomimetic mineralization in SBF, and lyophilization again, the MLM GCH microscaffolds were obtained. In the study, the non-mineralized GO-Col (NM GC) microscaffolds were fabricated by eliminating the biomimetic mineralization process and the other steps remained unchanged. The one-layer mineralized GO-Col-HAp (OLM GCH) microscaffolds were fabricated by mineralizing the outer layer (1500 μmφ), while the inner layer (800 μmφ) and middle layer (1200 μmφ) were not mineralized.

#### Fabrication of the PCL constructs

PCL constructs were fabricated by using the HTS Rapid Prototyping System. Briefly, PCL pellets were filled into the stainless steel nozzle (Temperature: 120 ℃, Pneumatic pressure: 670 kPa, fill rate: 10 mm/min). Cube-shaped (side length: 30 mm, height: 3 mm, line width: 800 μm, line height: 500 μm) PCL constructs were obtained.

#### Assembly of the microscaffolds into the PCL constructs

To assemble the microscaffolds into the PCL constructs, the cube-shaped PCL constructs were segmented into small pieces (5 mm inside length, 3 mm in height) using scissors. Then, three groups of microscaffolds (NM GC, OLM GCH, and MLM GCH) were packed into the pores of the PCL constructs. Each PCL construct was filled with 8 microscaffolds.

### Characterization of the microscaffolds and the assembled PCL constructs

#### Cross-section and pore morphologies and porosity evaluation

The cross-section and pore morphologies were observed under field emission scanning electron microscopy (FSEM, GeminiSEM300, German), and the pore diameters were quantitatively analyzed using image J software. The energy dispersive spectroscopy (EDS) was operated to characterize the Ca/P ratio and element distribution (including calcium and phosphorus elements). To measure the porosity of the microscaffolds, the dry microscaffolds were weighted (W_0_) and immersed in water for 12 h at room temperature to obtain the wet microscaffolds (weighted W_1_). The porosity was calculated by the equation:

$$\mathrm{Porosity }\left(\mathrm{\%}\right)=\left[({w}_{1}-{W}_{0})/\pi \rho h\left(d/2\right)\right]\times 100\%$$

$\rho$ is the density of the water 1.00 mg/mm^3^, $\pi$ is the circular constant 3.14159, d is the diameter of the microscaffold, and h is the height of the microscaffold.

#### Weight increase ratio and calcium quantitative analysis

To measure the weight increase ratio, the dry microscaffolds were weighted (W_d_) and immersed into SBF for 7 day at 37 ℃. The obtained mineralized microscaffolds were weighted (W_m_) again. The weight increase (%) was calculated by the equation:

$$\mathrm{Weight increase }\left(\mathrm{\%}\right)=\left[\left({W}_{m}-{W}_{d}\right)/{W}_{d}\right]\times 100\%$$

To analyze the calcium content in the microscaffolds after biomimetic mineralization, the mineralized microscaffolds were immersed in 0.5 mol/L acetic acid for 12 h. The calcium content was then calculated using a calcium assay kit (Jiancheng, Nanjing, China) according to the manufacturer’s instructions.

#### FTIR, XRD, Raman, and TGA analysis

To analyze the functional groups, FTIR spectroscopy (VERTEX 70, Bruker company, German) was performed from 4000 cm^−1^ to 500 cm^−1^ with a resolution of 0.4 cm^−1^ over 64 scans. To analyze the HAp crystalline phases, the X-ray diffractometer (XRD, Empyrean, PANalytical B.V.) was performed from 5° to 40° (2θ range) at a scanning rate of 0.013°s^−1^ with CuKα radiation (k = 1.540598 nm). To characterize the components of microscaffolds, a Raman spectrometer (LabRAM HR800, Horiba JobinYvo) was carried out at 532 nm for 10 s. To determine the amount of HAp in the microscaffolds, TGA (Diamond TG/DTA, PerkinElmer Instruments, China) was performed from 25 ℃ to 800 ℃ with a heating rate of 10 ℃/min in N_2_ atmosphere.

#### Elastic modulus analysis

The elastic modulus was analyzed by a compression test. The samples were put in the All-Electric Dynamic Test Instrument (ElectroPuls E1000, British) with a loading rate of 2 mm/min until 80% compression was reached. The stress–strain curves were obtained and the elastic modulus was obtained by fitting 0–10% of the curve and calculating the slope.

#### Water absorption and water retention

The water absorption (W_A_) and water retention (W_R_) were calculated as the following equations:

$$\mathrm{Water\,absorption }\left(\mathrm{\%}\right)=\left[\left({W}_{1}-{W}_{0}\right)/{W}_{0}\right]\times 100\%$$

$$\mathrm{Water\,retention }\left(\mathrm{\%}\right)=\left[\left({W}_{2}-{W}_{0}\right)/{W}_{0}\right]\times 100\%$$

W_0_ and W_1_ are the weights of dry and wetting microscaffolds, respectively. W2 is the weight of centrifuged (1000 rpm, 5 min) wetting microscaffolds.

### In vitro cellular evaluation of the microscaffolds

#### Isolation, culture of rat BMSCs

Rat bone marrow mesenchymal stem cells (rBMSCs) were isolated as described before [32]. Briefly, SD rats (3–5 days old) were euthanized by cervical dislocation and immersed in 75% alcohol solution for 15 min. Followed by separating the femurs and tibias from attached soft tissues. Then, the cartilages at the ends of the separated bones were cut off to expose the bone marrow cavities, subsequently flushing with culture medium until the cavities turned from red to white. Finally, the obtained bone marrow tissues were cultured with L-DMEM (containing 10% fetal bovine serum and 1% penicillin and streptomycin) at 37 ℃ and 5% CO_2_ atmosphere. The culture medium was changed every 3 days, and the r-BMSCs were passaged when the attached cells became confluent.

#### Seeding of rat BMSCs in microscaffolds

Passage 3 r-BMSCs (2 × 10^6^ cells) were re-suspended in 200 μl of culture medium, and the sterilized microscaffolds were placed in a 6-well plate. Then 50 μl of cell suspension (containing 5 × 10^5^ cells) was pipetted into the microscaffolds. After 2 h incubation, the cell-seeded microscaffolds were transferred to a new culture plate and cultured with L-DMEM at 37 ℃ and 5% CO_2_ atmosphere.

#### Adhesion, proliferation, and viability of rBMSCs in microscaffolds

For the cell adhesion evaluation, the unattached rBMSCs were carefully removed from the bottom of the plate and counted N. The cell adhesion was calculated by the equation:

$$\mathrm{Cell\,adhesion }\left(\mathrm{\%}\right)=\left[\left(5\times {10}^{5}-N\right)/5\times {10}^{5}\right]\times 100\%$$

5 × 10^5^ is the total amount of cells, and N is the number of cells attached to the plate.

The cell-seeded microscaffolds were placed in 96-well plates in advance. The proliferation of rBMSCs was evaluated using MTT assays at 1, 3, and 7 day postculture. The cell viability of rBMSCs seeded in microscaffolds was evaluated by live/dead cell imaging kit staining at 1, 3, and 7 day post culture. In brief, the samples were washed with phosphate-buffered saline (PBS) and incubated in 2 μM fluorescein diacetate (FDA, Sigma, staining live cells) for 30 min and 4 μM propidium iodide (PI, Sigma, staining dead cells) for 10 min at 37 ℃. The stained samples were finally washed with PBS and observed under a confocal laser microscope (Leica, Germany).

#### Osteogenic differentiation evaluation of rBMSCs in microscaffolds

Following a 14-day period of osteogenic differentiation, the corresponding microscaffolds were immobilized using 4% paraformaldehyde at ambient temperature, rinsed with distilled water, and subjected to ARS dye (G3281; Solarbio, China) treatment for a duration of 20 min. The extracellular calcium deposition was subsequently observed using an inverted microscope (TE300; Nikon, Japan). The cells were co-incubated with 10% acetic acid overnight, and the resulting supernatant was obtained via centrifugation and subsequently neutralized with 10% ammonium hydroxide. This was done to facilitate the quantification of ARS staining by measuring the absorbance of the supernatant at a wavelength of 405 nm.

The rBMSCs were cultured on microscaffolds for a period of 14 days in an osteoinductive culture. Following this, the total protein of the rBMSCs was extracted from the microscaffolds. Subsequently, 40 μg of protein was loaded onto a 10% SDS/PAGE gel and transferred to a PVDF membrane. After blocking, the PVDF membranes were treated with rabbit runt-related transcription factor 2 (Runx2), alkaline phosphatase (ALP), collagen type-1 (Col-1), osteocalcin (OCN) polyclonal antibodies (Abcam, USA), and glyceraldehyde 3-phosphate dehydrogenase (GAPDH) monoclonal antibody (Sigma, USA), respectively at a temperature of 4 ℃ overnight. The bands were then incubated with horseradish peroxidase (HRP)-conjugated secondary antibodies at room temperature for a duration of 2 h. The targeted proteins were visualized using ECL reagents and quantified using Image J software. qRT-PCR was used to analyze osteogenesis differentiation of rBMSCs cultured on/in microscaffolds for 14 day. The targeted miRNAs were Runx2, ALP, OCN, and Col-1. In this study, the cells were subjected to a washing step using D-PBS, followed by the extraction of total RNA from rBMSCs cultured on microscaffolds using Trizol (TaKaRa). Subsequently, cDNA synthesis was performed using 1 µg of RNA and a RevertAid First Strand cDNA Synthesis Kit (TaKaRa). Furthermore, the cDNA was amplified using a qRT-PCR assay, employing the SYBR Premix Ex Tag Kit (TaKaRa) and an ABI 7500 Sequencing Detection System (Applied Biosystems). The primer sequences were listed in Supporting Information Additional file 1: Table S1.

The rBMSCs were seeded on microscaffolds cultured for 14 day osteoinductive culture and then the total protein of rBMSCs was extracted from the microscaffolds. A total of 40 μg protein was loaded onto 10% SDS/PAGE gel and then transferred to PVDF membrane (Millipore, USA). After blocking, the pre-treated PVDF membranes were incubated with rabbit runt-related transcription factor 2 (Runx2), alkaline phosphatase (ALP), collagen type-1 (Col-1), osteocalcin (OCN) polyclonal antibodies (Abcam, USA), and glyceraldehyde 3-phosphate dehydrogenase (GAPDH) monoclonal antibody (Sigma, USA), respectively at 4 ℃ overnight. Then the bands were incubated with horseradish peroxidase (HRP)-conjugated secondary antibodies at room temperature for 2 h. The targeted proteins were visualized using ECL reagents and quantified using image J software. qRT-PCR was used to analyze osteogenesis differentiation of rBMSCs cultured on/in microscaffolds for 14 day. The targeted miRNAs were ALP, OCN, and Col-1, while GAPDH was used as an internal control for mRNAs. The primer sequences were listed in Supporting Information Additional file 1: Table. S1.

### In vivo evaluation of the assembled PCL constructs on bone defect

30 male SD rats (4–6 weeks old, 180-220 *g* weight) were randomly divided into five groups: (1) Blank; (2) PCL; (3) NM GC/PCL; (4) OLM GCH/PCL; (5) MLM GCH/PCL (n = 6 rats per group). The SD rats were anesthetized with 3% pentobarbital sodium. 2 cm length incision was cut on the right cheek, and the muscle and fascia were cut open to expose the mandible. Followed by drilling a 5 mm mandibular defect, filling the samples, and closing the incision. After feeding for 4 weeks and 12 weeks, the SD rats were sacrificed and isolated from the mandibles. The obtained samples were fixed in 10% formalin and scanned using a Micro-CT scanner (SkyScan 1176, Broker). 3D reconstruction images were performed using Mimics Research software. New bone volume relative to tissue volume (BV/TV) and bone mineral density (BMD) were calculated using CTAn software.

### Histology and immunohistochemical staining

The obtained samples were decalcified in 10% ethylene diamine tetraacetic acid (EDTA) for 4 weeks, embedded in paraffin, and cut into histological slices (3 μm thickness). Then, the hematoxylin and eosin (HE) and Masson’s trichrome were used to stain the histological slices. For immunohistochemical analyses, the slices were blocked by diluted ghost serum antibody, incubated with OCN monoclonal antibody (Abcam, UK), and observed under the microscope.

### Statistical analysis

All data presented in the experiments are shown as the mean ± SD. Each experiment was repeated at least three times. Student’s t-test was used for two groups comparing. One-way analysis of variance (ANOVA) was applied for multiple-group comparisons. P < 0.05 was considered to be a statistically significant difference.

## Results

### Morphology and structural evaluation of microscaffolds after biomimetic mineralization

**Fig. 2:**
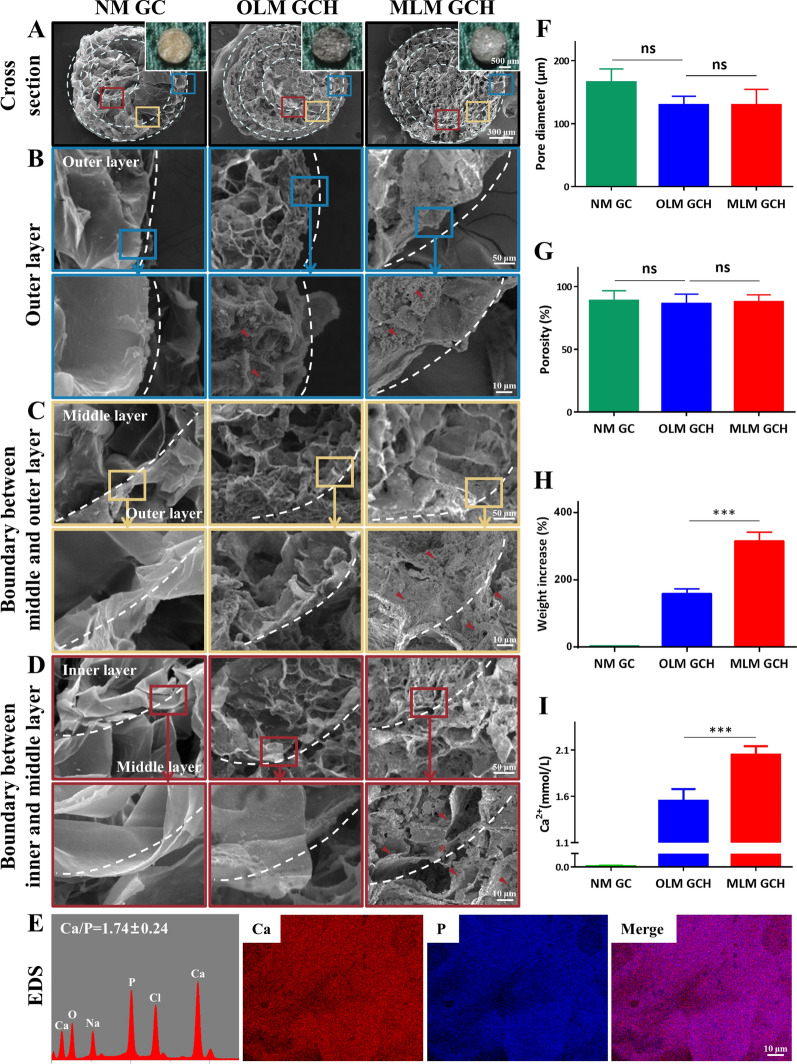
Structural characterization of the microscaffolds. **A** Optical and SEM images of the cross-section of the microscaffolds. The scale bars are 500 μm in optical images and 300 μm in SEM images. **B**–**D** SEM images of the corresponding outer layer, middle layer, and inner layer of the microscaffolds. The scale bars are 50 μm in low magnification and 10 μm in high magnification of SEM images. **E** EDS point and mapping analysis of the MLM GCH microscaffolds. The scale bar is 10 μm. **F** The pore diameter of the microscaffolds. **G** The porosity of the microscaffolds. **H** Weight increase of the microscaffolds after 7 days of mineralization. **I** Calcium ion concentrations of the microscaffolds after dissolving the HAp in acetic acid. ***p < 0.001, and *ns* no significance

For a comprehensive assessment of the microscaffold structure, cross-sections were obtained by crosscutting, revealing distinct features **(**Fig. 2A**)**. The NM GC microscaffold displayed a brown hue, whereas the OLM GCH and MLM GCH microscaffolds exhibited black coloring. Notably, white crystals (indicated by white arrowheads) were visible in the cross-section of MLM GCH microscaffolds. SEM microscopy revealed porous, interconnected, and multi-layered structures (dotted circles) across all microscaffold groups. SEM analysis revealed that the pore diameters were 165.7 ± 21.1 μm for NM GC, 129.7 ± 13.9 μm for OLM GCH, and 129.3 ± 25.1 μm for MLM GCH **(**Fig. 2F**)**. Microscaffold porosity was also examined (88.7 ± 8.0% for NM GC, 86.3 ± 7.6% for OLM GCH, and 87.7 ± 5.7% for MLM GCH), revealing no significant differences (Fig. 2G). A more detailed analysis of HAp distribution within microscaffolds post-mineralization was conducted at different layers (outer layer in blue rectangle, middle layer in yellow rectangle, inner layer in red rectangle). The uniform distribution of HAp crystals (indicated by red arrowheads) was evident across the three layers of MLM GCH microscaffolds. In contrast, HAp was only deposited in the outer layer of OLM GCH microscaffolds, while NM GC microscaffolds showed no HAp distribution (Fig. 2B–D). Characterization of crystals within the inner layer of MLM GCH microscaffolds was achieved through EDS point analysis and mapping (Fig. 2E**)**. The Ca/P ratio of these crystals was 1.74 ± 0.24, approximating that of natural bone HAp. EDS mapping images displayed similar Ca and P element distribution, consistent with SEM results. To quantify HAp deposition, weight increase ratios were calculated **(**Fig. 2H**)**, with the MLM GCH group exhibiting significantly higher ratios (314.6 ± 26.8%) than the OLM GCH group (157.8 ± 15.3%). A similar trend was noted in calcium quantitative analysis (Fig. 2I), as the MLM GCH group showed elevated calcium ions (2.05 ± 0.09 mmol/L) compared to the OLM GCH group (1.553 ± 0.13 mmol/L).

### Physiochemical and mechanical evaluation of the microscaffolds

**Fig. 3:**
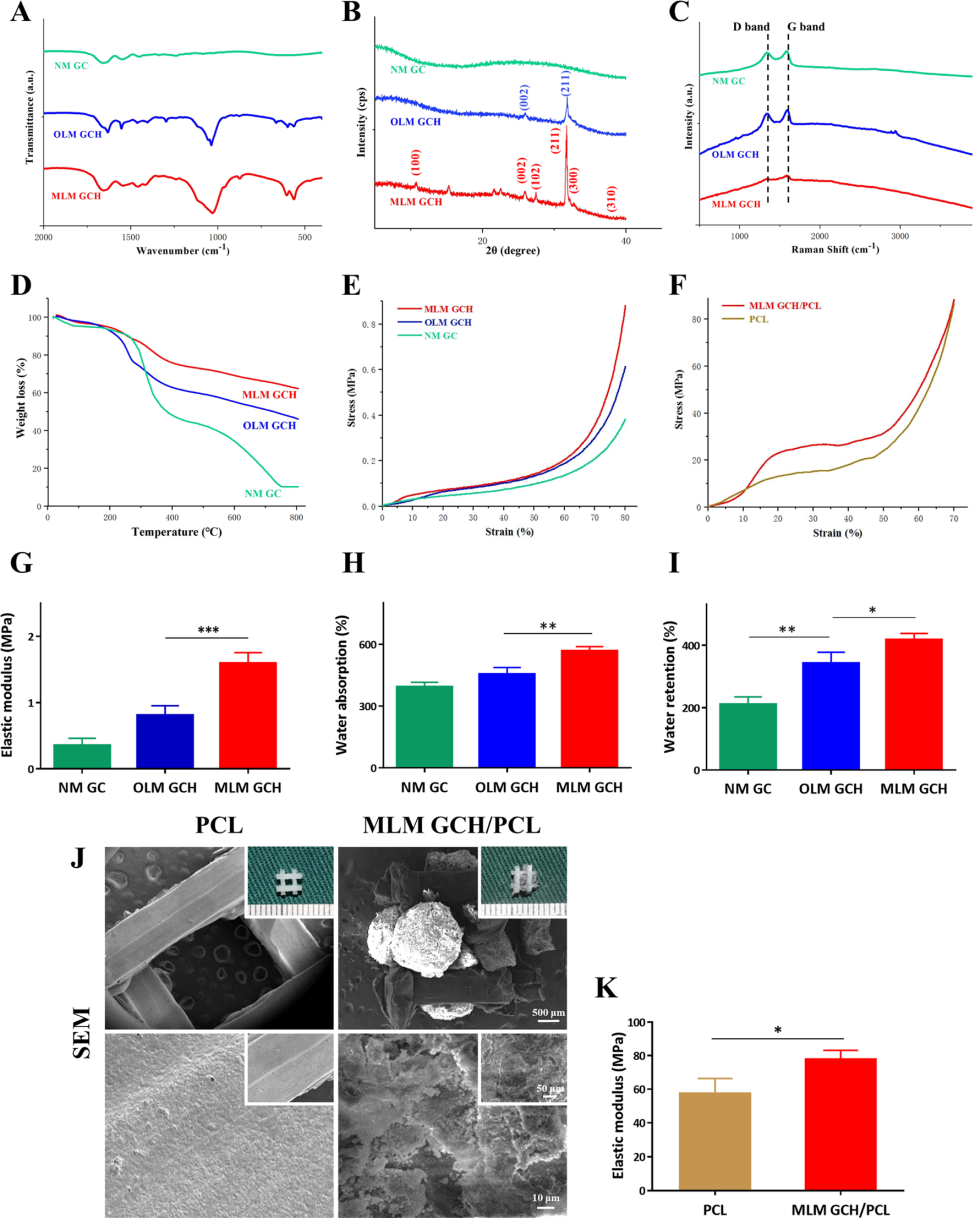
Physicochemical characterization of the microscaffolds. **A** FT-IR spectrum, **B** XRD, **C** Raman spectrum, **D** TGA analysis, and mechanical testing of the microscaffolds **E**, **G** and PCL assembled microscaffolds **F**, **K**. **H** Water absorption of the microscaffolds. **I** Water retention of the microscaffolds. **J** Optical and SEM images of the PCL scaffold and assembled MLM GCH/PCL scaffolds. The scale bars are 500 μm in low magnification, 50 μm in middle magnification, and 10 μm in high magnification of SEM images. *p < 0.05, **p < 0.01, and ***p < 0.001

To confirm the chemical composition of the microscaffolds, FTIR spectra analysis was performed **(**Fig. 3A**)**. Peaks at 1645 cm^−1^ (amide I C = O stretch) and 1566 cm^−1^ (amide II N–H deformation) suggested amide bonds (-NHCO-) formed via chemical crosslinking [32]. Mineralized microscaffolds displayed absorption peaks at 1025 cm^−1^, 960 cm^−1^, 600 cm^−1^, and 558 cm^−1^, corresponding to PO4^3−^ group vibrations of apatite [33]. The crystalline structure of mineralized apatite was verified by XRD measurements (Fig. 3B), with the MLM GCH group revealing peaks corresponding to HAp (100), (002), (102), (211), (300), and (310) diffraction peaks [34]. In contrast, mineralized apatite in the OLM GCH group exhibited only two peaks (at 25.8° and 31.9°), indicating reduced crystallinity. Additionally, a sharp and prominent peak was observed at 2θ = 31.8° in the MLM GCH group, attributed to enhanced crystal growth at the (211) reflection of HAp. Raman spectrum analysis was conducted to identify microscaffold components **(**Fig. 3C**)**. Two peaks at 1354 cm^−1^ and 1598 cm^−1^, corresponding to the D band and G band, indicated the successful incorporation of GO into the microscaffolds [35]. The intensity of the D and G band peaks was lower in the MLM GCH group due to interactions between GO and HAp that reduced lattice defects of the GO bands [36]. TGA analysis confirmed changes in HAp mass **(**Fig. 3D), with remaining weights of NM GC, OLM GCH, and MLM GCH groups at 10.7%, 46.8%, and 61.7%, respectively, indicating higher HAp deposition in MLM GCH microscaffolds.

Mechanical properties were assessed via compression testing (Fig. 3E, G). Stress–strain curves were prepared using Origin software, revealing higher stress in the MLM GCH group (Fig. 3E). Elastic modulus was calculated from the 0–10% curve region, with the MLM GCH group exhibiting a higher elastic modulus (1.59 ± 0.17 MPa) compared to the other two groups (0.81 ± 0.14 MPa for OLM GCH group, 0.35 ± 0.11 MPa for NM GC group) **(**Fig. 3G**)**. The PCL scaffold and assembled PCL construct elastic moduli were also assessed (Fig. 3F, J, K). Due to the robust mechanical properties of PCL scaffolds (28.67 ± 4.54 MPa), the assembled PCL constructs had an increased elastic modulus of 38.83 ± 4.54 MPa **(**Fig. 3J, K**)**.

It is well-established that water absorption and retention rates are linked to nutrient transfer and cell proliferation. Mineralized groups demonstrated higher water absorption rates than non-mineralized NM GC microscaffolds (455.1 ± 32.8% for the OLM GCH group, 566 ± 22.5% for the MLM GCH group) **(**Fig. 3H**)**. Furthermore, elevated water retention rates were observed in the OLM GCH group (343.2 ± 35.0%) and MLM GCH group (418 ± 20.0%) compared to the NM GC group (210.7 ± 23.5%) **(**Fig. 3I**)**.

### In vitro analysis of microscaffolds on cell adhesion, proliferation, and differentiation

**Fig. 4:**
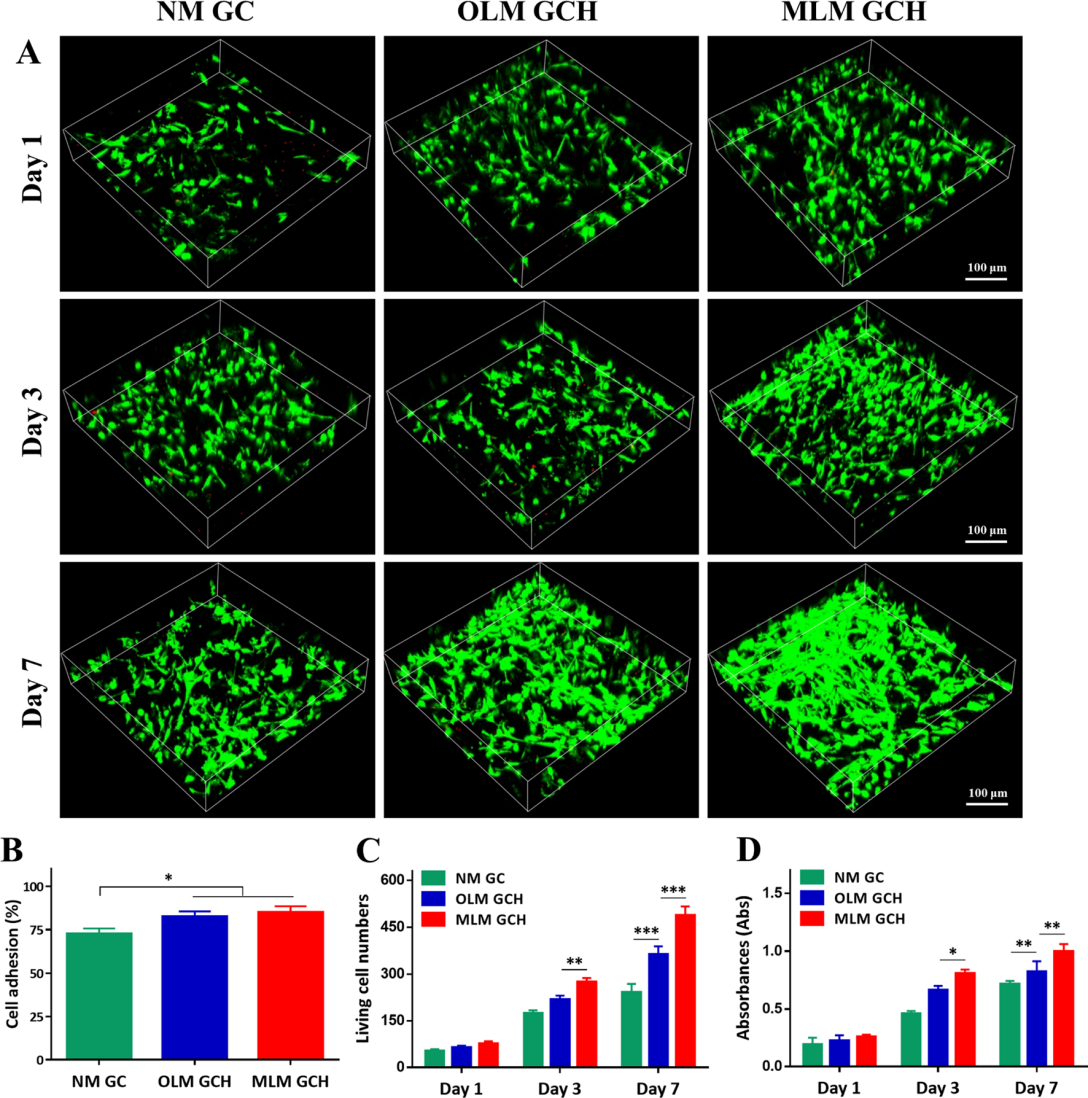
In vitro cellular evaluation of the microscaffolds. **A** Live/dead staining of rBMSCs in the microscaffolds after 1 day, 3 day, and 7 day incubation. **B** The cell adhesion ability of the rBMSCs seeded in the microscaffolds. **C** Quantitative analysis of the living cells in the microscaffolds. **D** MTT analysis of the rBMSCs in the microscaffolds after 1 day, 3 day, and 7 day incubation. *p < 0.05, **p < 0.01, and ***p < 0.001

For the assessment of cell viability within microscaffolds, live/dead staining and confocal laser microscopy were employed on days 1, 3, and 7 post-cell seeding (Fig. 4A). Enhanced presence of viable cells (stained green) was evident in the OLM GCH group and MLM GCH group, predominantly distributed within the microscaffolds rather than the NM GC scaffolds. These observations were consistent with the outcomes of cell adhesion experiments, where the MLM GCH group demonstrated a superior cell adhesion rate (84.97 ± 3.57%) compared to the other two groups (72.53 ± 3.26% for NM GC group, and 82.67 ± 2.94% for OLM GCH group) (Fig. 4B). Upon extending the incubation period to 7 days, the MLM GCH group maintained a higher population of viable cells, uniformly dispersed within the MLM GCH microscaffolds. Quantitative analysis of live cells from live/dead staining images substantiated these findings, demonstrating a greater distribution of viable cells in the MLM GCH groups on days 3 and 7 post-cell seeding (Fig. 4C). The proliferation of cells was assessed through MTT assay on days 1, 3, and 7 post-seeding (Fig. 4D). Substantial increases in proliferation were noted across all groups with extended incubation time. Interestingly, the MLM GCH group exhibited the highest proliferation rate on days 3 and 7, consistent with the outcomes of the live/dead staining experiments.

**Fig. 5:**
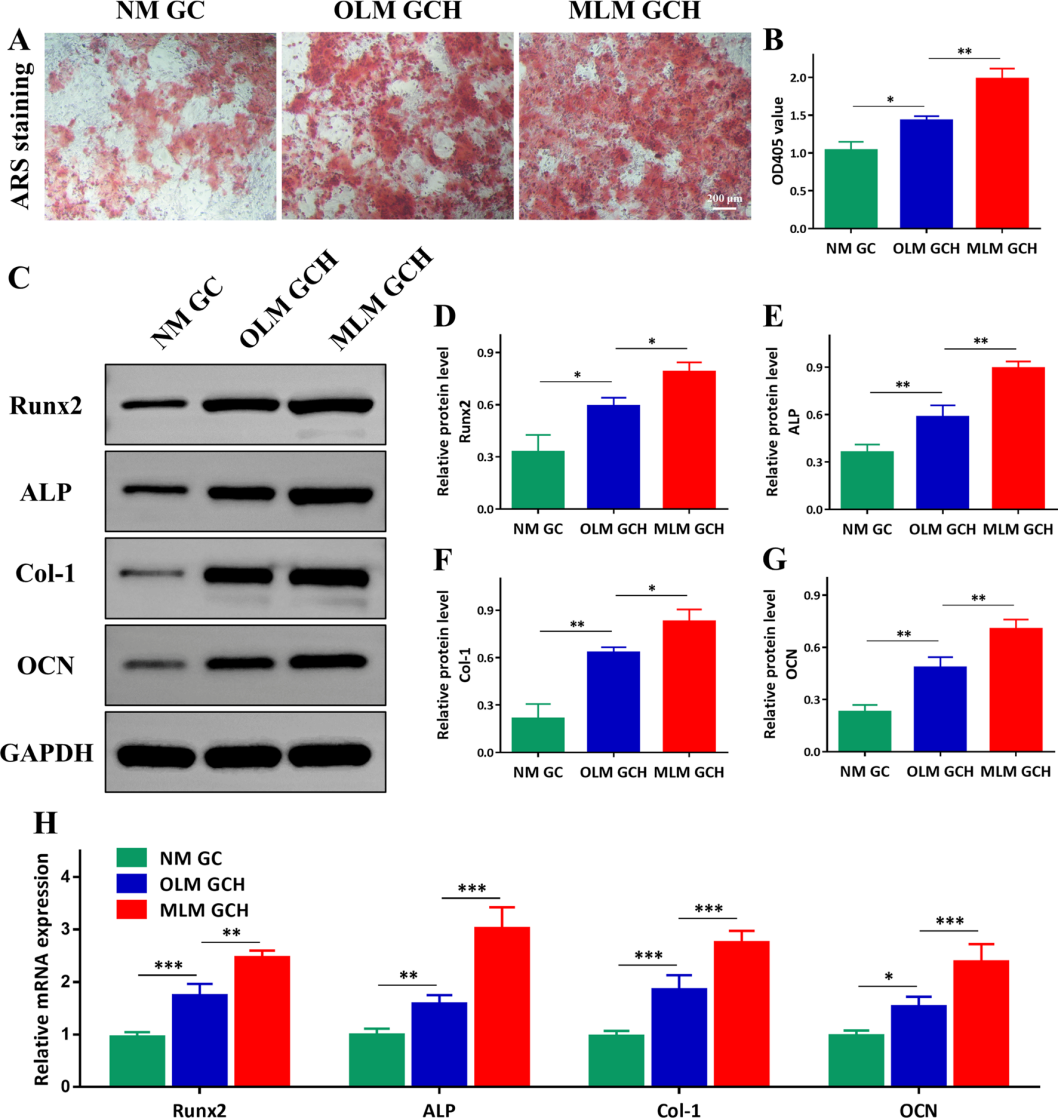
In vitro osteogenic differentiation evaluation of the microscaffolds. **A** ARS staining following 14 days of incubation with rBMSCs. Scale bar: 200 μm. **B** Quantitative analysis of ARS staining. **C** Western blot evaluation of osteogenic-related protein expression. **D**–**G** Quantitative data for Runx2, ALP, Col-1, and OCN respectively. **H** Osteogenic-related gene analysis of rBMSCs on different microscaffolds. *p < 0.05, **p < 0.01, and ***p < 0.001

The osteogenic potential of tissue-engineered materials plays a crucial role in the healing and regeneration of bone defects. As depicted in Fig. 5A, the presence of calcium nodules in the NM GC, OLM GCH, and MLM GCH groups exhibited a gradual increase following alizarin red staining, with the MLM GCH group demonstrating significantly stronger calcium nodules compared to the other two groups. Furthermore, the quantitative relative values obtained through a 405 nm microplate reader were consistent with the staining outcomes (Fig. 5B). The Western blot analysis revealed significant upregulation of osteogenic-related proteins Runx2, ALP, Col-1, and OCN in the MLM GCH group compared to the other two groups (Fig. 5C). The quantitative analysis of the expressed proteins further confirmed these findings (Fig. 5D–G). Furthermore, the gene expressions of Runx2, ALP, Col-1, and OCN were evaluated using qRT-PCR assays (Fig. 5H). Overall, the results demonstrated that the MLM GCH group exhibited superior osteo-inductive ability compared to the other two groups, as evidenced by alizarin red staining and western blot analysis.

### In vivo analysis of the assembled PCL constructs on mandibular bone regeneration

**Fig. 6:**
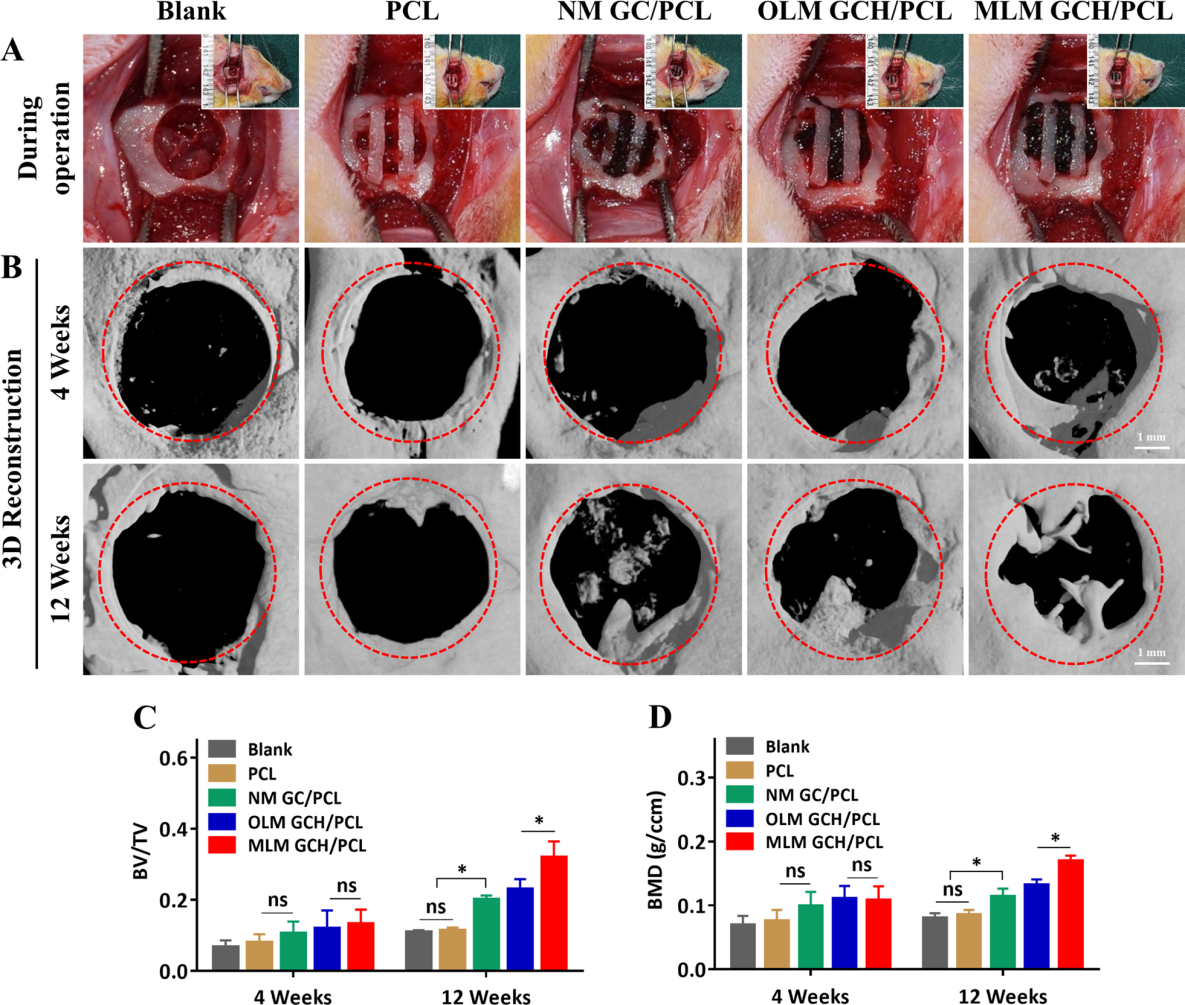
In vivo evaluation of the PCL and assembled microscaffolds for critical-sized mandibular bone defects treatment in the rat. **A** Optical images of the assembled microscaffolds in the mandibular defects during the surgery. **B** After 4 and 12 weeks of implantation, the defect regions were reconstructed by the micro CT. Scale bars: 1 mm. **C**, **D** The quantitative analysis of BV/TV (**C**) and BMD (**D**). *p < 0.05, and *ns* no significance

The osteogenic capabilities of PCL, NM GC/PCL, OLM GCH/PCL, and MLM GCH/PCL scaffolds were assessed in rat mandibular bone defects (Fig. 6A). Micro-CT imaging was employed to evaluate newly formed bone at 4- and 12-weeks post-implantation. 3D reconstructed images were generated, and the defect regions were highlighted with red dotted circles **(**Fig. 6B**)**. A limited amount of new bone growth was observed along the defect margins in all groups at 4 weeks post-implantation. At 12 weeks, the MLM GCH/PCL group displayed more substantial and densely formed bone tissue than the other groups. These newly generated bone tissues were distributed both centrally and at the periphery of the defect region. Quantitative analysis of BV/TV in the MLM GCH/PCL group (32.0 ± 4.4%) at 12 weeks post-implantation showed a significant increase compared to the OLM GCH/PCL (23.0 ± 2.8%), NM GC/PCL (20.1 ± 1.1%), PCL (11.3 ± 0.9%), and Blank (11.0 ± 0.5%) groups (Fig. 6C). BMD analysis **(**Fig. 6D) yielded similar outcomes, with the MLM GCH/PCL group (0.169 ± 0.008 *g*/ccm) displaying the highest BMD compared to other groups (0.132 ± 0.008 for OLM GCH/PCL group, 0.114 ± 0.012 for NM GC/PCL group, 0.085 ± 0.008 for PCL group, and 0.080 ± 0.008 for Blank group).

**Fig. 7:**
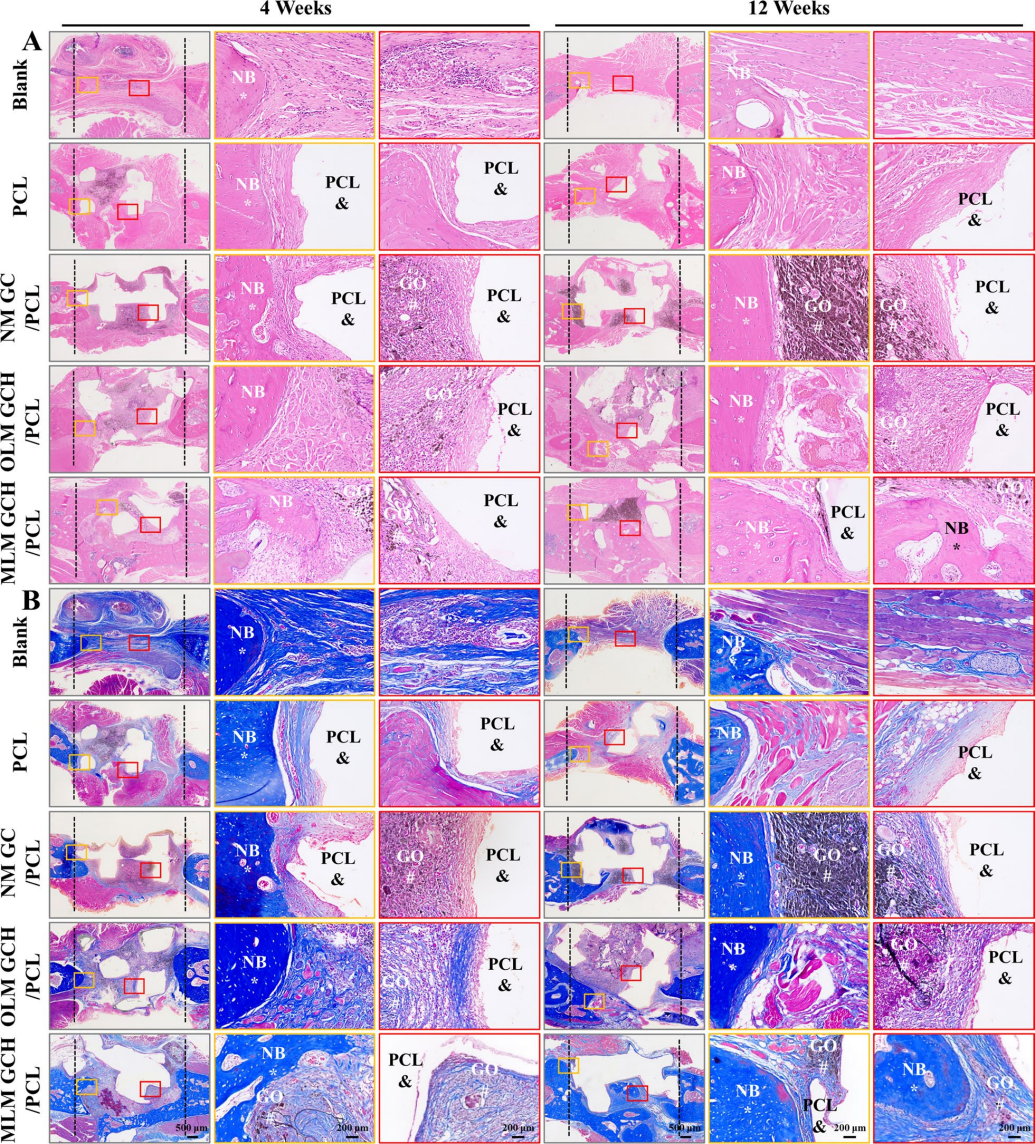
Histological evaluation of the mandibular defect regions after 4 and 12 weeks of implantation. **A** HE staining and **B** Masson staining of the mandibular defect regions. & refers to PCL scaffold, * refers to new bone (NB), and # refers to GO. The scale bars in the low magnification images are 500 μm, and in the high magnification images are 200 μm

**Fig. 8:**
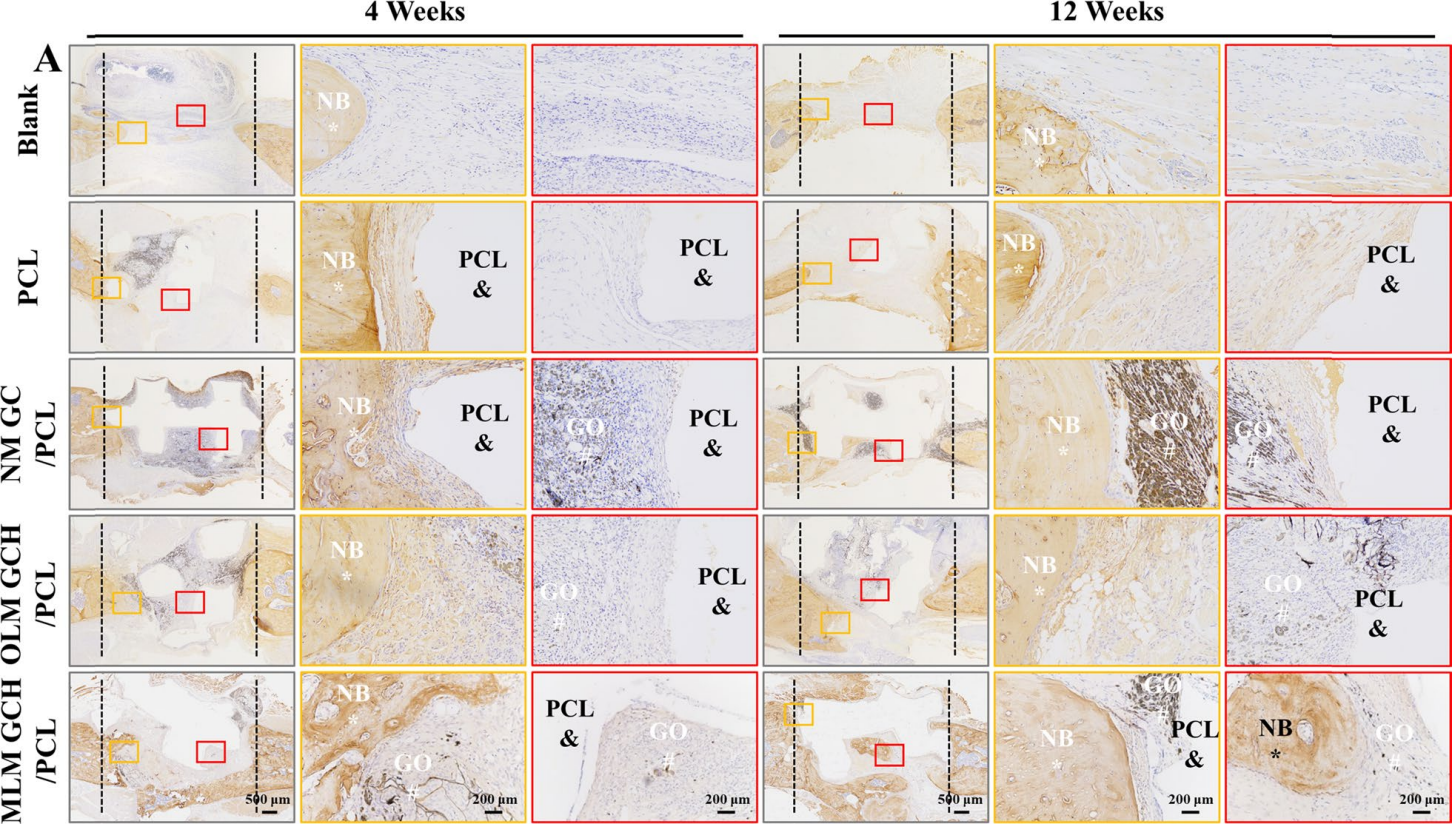
Immunohistochemical staining of the samples after 4 and 12 weeks of implantation. & refers to PCL scaffold, * refers to new bone (NB), and # refers to GO. The scale bars in the low magnification images are 500 μm, and in the high magnification images are 200 μm

The histological assessment involved HE staining to visualize assembled PCL constructs and newly formed bone tissues (Fig. 7A). At 4 weeks post-implantation, no evident necrosis or fibrous membranes were observed across all groups. PCL scaffolds (Fig. 7A, indicated by &) were evident in all groups, while newly formed bone tissues (Fig. 7A, indicated by *) were situated at the lower regions of the defect. After 12 weeks of implantation, the MLM GCH/PCL group exhibited more mature and densely formed bone tissue than other groups. Notably, these newly formed bone tissues bridged the scaffolds with the native bone, extending into the core of the PCL scaffolds (Fig. 7A). Masson staining was utilized to assess type I collagen formation (stained blue) within the defect regions. Similar observations were made, indicating the absence of substantial fibrous membranes surrounding the scaffolds at 4 weeks post-implantation (Fig. 7B). As the implantation period progressed to 12 weeks, the MLM GCH/PCL group displayed more mature and densely formed bone tissue than other groups (Fig. 7B, S1A, B). To further explore the osteogenic potential of the scaffolds, immunohistochemical staining for OCN expression was conducted (Fig. 8A). After 4 weeks of implantation, the positive staining (brown) new bone tissues in the MLM GCH/PCL group was relatively higher than in other groups. When the time prolonged to 12 weeks, more new bone tissues appeared in the MLM GCH/PCL group, showing more positive staining tissues and distributing within the bone defect (Fig. 8A). The quantitative analysis of the brown areas confirmed that the MLM GCH/PCL group exhibited the highest positive areas among other four groups at 12 weeks (Additional file 1: Fig. S2A).

## Discussion

The remarkable architecture and properties of natural bone provide a blueprint for developing advanced organic–inorganic composite materials that mimic its extracellular matrix and exhibit superior mechanical characteristics. In this study, we innovatively crafted multi-layer mineralized GO-Col-HAp microscaffolds using freeze-drying techniques and successive mineralization in simulated body fluid. These microscaffolds were subsequently integrated into a three-dimensional-printed PCL framework. The resulting composite MLM GCH/PCL scaffolds exhibited interconnected porous structures, boasting ample surface area and complete mineralized hydroxyapatite layers. The integration of the multi-layer mineralized configuration with the 3D-printed PCL framework engendered favorable mechanical attributes. Notably, these microscaffolds exhibited a propensity to enhance the adhesion, proliferation, and osteogenic differentiation of rat bone marrow stromal cells (rBMSCs) in vitro. Most importantly, these scaffolds displayed commendable bone regeneration capabilities in a rat mandibular bone defect model.

Over the past decade, 3D printing technologies have emerged as a promising manufacturing approach, particularly in bone graft substitutes, owing to their favorable mechanical properties, customizable pore geometry, and tailored structures [37]. Leveraging CT or MRI imaging data alongside computer-aided design (CAD) models, 3D printing ensures reproducibility, accuracy, and controlled microstructure design for bone graft substitutes [38]. Among the various 3D printing techniques (such as SLA, SLS, and FDM), FDM stands out for its maturity, broad applications, rapid processing, and cost-effectiveness. Moreover, FDM's straightforward, versatile, and solvent-free process mitigates safety concerns associated with certain organic solvents [39]. The present study considers the biodegradable polymer/bioceramic composite as a viable scaffold material in the field of bone tissue engineering (BTE) [40]. This is due to the fact that incorporating a polymer phase into a porous ceramic scaffold enhances the fracture toughness of the composite and facilitates surface functionalization, thereby promoting improved bioactivity. In this investigation, polycaprolactone (PCL) was selected as the polymer framework owing to its biodegradability, favorable biocompatibility, absence of inflammatory response, and the added benefit of being approved by the FDA for use in implant applications [41]. Additionally, FDM technique was employed to fabricate the 3D PCL framework featuring interconnected cube-shaped structures (Fig. 3J). These designed pore sizes and interconnected structures in the PCL scaffolds were well-suited for assembling microscaffolds. Their adequate mechanical properties **(**28.67 ± 4.54 MPa for PCL scaffolds, Fig. 3F, K) assured the scaffolds' capacity for bone regeneration by withstanding the pressures exerted around the bone defect. Furthermore, the interconnected design ensured efficient nutrient exchange and cell growth within the microscaffolds **(**Fig. 4**)**.

Col has been widely utilized as scaffolds in bone regeneration due to its excellent biocompatibility and easy modification. The poor mechanical properties limit its applications in bone repair [18]. Although several strategies have been attempted to reinforce their mechanical strength, satisfactory strength for bone regeneration was still unachieved [29]. In this study, GO, which is acknowledged as a promising nanomaterial to improve the mechanical properties of scaffolds, was employed to enhance the mechanical properties of Col. Furthermore, due to the abundant functional groups of hydroxyl and carboxyl groups on the surface of GO [35], the GO functionalized Col scaffolds were employed as a substrate to absorb the bioactive ions. The bioactive ions, including calcium and phosphate ions, coalesced to form the nucleate and subsequently developed into hydroxyapatite (HAp). Furthermore, the π-π bonds present on the graphene oxide (GO) facilitated the adsorption of proteins and cells in the vicinity. Consequently, the combined action of GO and mineralized HAp resulted in enhanced cell proliferation and osteogenic differentiation, as illustrated in Figs. 4 and 5. The fabricated GO-Col-HAp microscaffolds exhibit great potential as scaffolds in bone tissue engineering.

Natural bone extracellular matrix, a composite of collagen and hydroxyapatite, presents a hierarchically staggered structure and serves as a model for desirable bone tissue regeneration [42]. A novel multi-layer mineralized GO-Col-HAp microscaffold was engineered to replicate this ECM architecture. The fabrication process began with the initial integration of GO and Col to establish the microscaffold’s foundation, followed by hydroxyapatite deposition through biomimetic mineralization in SBF. Constructing a 3D microscaffold with nano- and microarchitecture involved the freeze-drying method to establish a porous, interconnected collagen-based microstructure. Inspired by the organic template-driven mineralization process in bone formation, the collagen-based microscaffolds underwent biomimetic mineralization in SBF to emulate nano-hydroxyapatite formation within collagen fibers. In contemporary times, HAp has gained significant popularity as a bone substitute material due to its resemblance to the inorganic component of human and animal bones, favorable biocompatibility, osteoconductivity, and osteoinductivity [43]. The crystalline structure and morphology of HAp play crucial roles in modulating cellular behaviors [44]. Additionally, HAp facilitates expedited bone regeneration and direct integration with regenerated bones, bypassing the need for intermediate connective tissues. Although HAp formed by SBFs is often poorly distributed in the central area of some large stents or three-dimensional (3D) complex stents, we employed the multi-layered structure to harvest the uniform distribution of HAp on the microscaffold’s surface and the inner regions (Fig. 2A–D). The MLM GCH microscaffolds were engineered to replicate the composition and fibrillar architecture of the ECM through the utilization of these specific materials of collagen and mineralized hydroxyapatite. The freeze-drying method and multi-layer mineralized process were employed to create a porous, hierarchical, and interconnected microstructure that emulates the nano- and micro-hierarchical architecture of the ECM. The incorporation of GO was implemented to augment the biomineralization process in the Col and stimulate cell proliferation and osteogenic differentiation, thereby imitating the biological characteristics of the native ECM [29]. This sophisticated nano- and microstructure bestowed the fabricated ECM-inspired multi-layer mineralized GO-Col-HAp microscaffold with favorable attributes, including suitable mechanical strength, effective water absorption and retention, and favorable cell compatibility (Figs. 3D–H, 4A–D).

Mimicking the composition and micro-nano structure of natural ECM is beneficial to bone tissue regeneration [45]. To verify the bone regeneration effect of bone ECM-inspired assembling microscaffolds, a model involving a critically-sized mandibular bone defect known to be challenging for natural clinical self-repair was utilized [3]. The mandibular defect model is frequently employed in dental applications [46]. The ramus, a mandibular subunit responsible for bearing dynamic loads during mastication, is subjected to additional challenges through the creation of a defect between the masseter and medial pterygoid muscles, which are promptly activated following anesthesia [47]. Consequently, the repair and regeneration of these defects present considerable challenges due to the intricate nature of the involved structures and the complex biomechanical and physiological environment [2]. In this context, the MLM GCH/PCL group exhibited more densely formed new bone tissue than the other groups. These new bone tissues were distributed both in the central and marginal regions of the defect (Figs. 6, 7, 8). The mechanisms underlying this in vivo bone regeneration process are multi-faceted. In total, the innovative assembly of these graphene oxide, collagen, mineralized hydroxyapatites, and 3D-printed PCL into a biomimetically hierarchical scaffold contributed to enhanced bone regeneration effects. 3D-printed PCL framework with macro-porous architecture was employed as a supportive substrate for mandibular defect repair. Additionally, novel multi-layer mineralized and hierarchical designed GO-Col-HAp microscaffolds were fabricated to emulate the physiochemical and biological properties of native ECM. The nano- and microstructure of the microscaffold provided a porous, interconnected, and multi-layer mineralized environment conducive to host cell ingrowth, proliferation, and differentiation. Upon placement of the microscaffold at the defect site, the PCL framework offered mechanical support, enabling host-derived cells to infiltrate the microscaffold rapidly. Guided by the multi-layer mineralized nano-hydroxyapatite, these host cells underwent continuous proliferation and differentiation, culminating in new bone formation.

While graphene oxide exhibits substantial potential as an inorganic material for bone tissue engineering, concerns remain regarding its biotoxicity [48, 49]. Studies have shown that GO’s potential biotoxicity is closely linked to factors such as structure, concentration, size, and degree of functionalization [50]. In the study, GO was observed in the HE and Masson staining as a dark network structure, and there was no obvious inflammatory cell infiltration around GO at 4 and 12 weeks (Fig. 7). The negligible biotoxicity and good biocompatibility of GO-based microscaffolds benefited from the following factors. Firstly, the GO concentration in the research was 1 mg/mL, significantly lower than the toxic concentration reported in the literature [32]. Secondly, GO within the microscaffold formed chemical cross-links with collagen and absorbed nano-hydroxyapatite, reducing potential tissue damage and minimizing biotoxicity [51]. Moreover, the implanted GO-based microscaffolds within the defect region could undergo metabolization through macrophage-mediated phagocytosis via HRP and MPO, eventually being excreted via the lungs and kidneys [52].

## Conclusions

In summary, the assembly strategy of 3D-printed PCL frameworks with ECM-inspired and multi-layer mineralized GO-Col-HAp microscaffolds has yielded promising outcomes. The PCL framework furnished requisite mechanical strength and assembly space, while the multi-layer mineralized GO-Col-HAp microscaffolds emulated natural bone ECM in terms of nano- and microstructure. These microscaffolds boasted interconnected, porous microarchitecture and featured hierarchically distributed nano-HAp characteristics that facilitated efficient nutrient exchange, water absorption, water retention, and support cell adhesion and proliferation in vitro. Crucially, these assembled scaffolds demonstrated robust bone regeneration efficacy in critical-sized mandibular bone defects. Therefore, the integration of 3D-printed PCL frameworks and ECM-inspired microscaffolds holds significant promise for promoting bone regeneration and has potential applications in various biomaterial assembly contexts.

### Supplementary Information

**Additional file 1: Fig. S1.** Quantitative analysis of new bone area in HE and Masson staining. **Fig. S2.** Quantitative analysis of the expression level of OCN. **Table S1.** Primer sequences used for gene expression analysis by qRT-PCR.

## Data Availability

All data generated or analyzed during this study are included in this published article.

## Abbreviations

PCL: Polycaprolactone
GO: Graphene Oxide
Col: Collagen
ECM: Extracellular matrix
SBF: Simulated body fluid
HAp: Hydroxyapatite
OCN: Osteocalcin
HE: Hematoxylin Eosin
BMSC: Bone marrow mesenchymal stem cell
MTT: 3-(4,5-Dimethylthiazol-2-yl) -2,5-diphenyltetrazolium bromide
PBS: Phosphate Buffer Saline
SEM: Scanning electron microscopy
XRD: X-ray Power Diffraction
FDA: Fluorescein diacetate
PI: Propidium iodide
DMSO: Dimethyl Sulfoxide
FBS: Fetal bovine serum
L-DMEM: Low-glucose Dulbecco’s modified eagle medium
