# Supplementary material for: 3D-printed PCL framework assembling ECM-inspired multi-layer mineralized GO-Col-HAp microscaffold for in situ mandibular bone regeneration
Source: J Transl Med. 2024 Mar 1;22:224. doi: 10.1186/s12967-024-05020-1 (PMC10908055; doi:10.1186/s12967-024-05020-1)
Supplement: Supplementary file 1 — Additional file 1: Fig. S1. Quantitative analysis of new bone area in HE and Masson staining. Fig. S2. Quantitative analysis of the expression level of OCN. Table S1. Primer sequences used for gene expression analysis by qRT-PCR. [file 12967_2024_5020_MOESM1_ESM.zip › New folder/Supplementary Material.docx]

Supplementary Material

**3D-printed PCL framework assembling ECM-inspired multi-layer mineralized**

**GO-Col-HAp microscaffold for in situ mandibular bone regeneration**

Yanqing Yang,^a,^‡ Huan He,^c,^‡ Fang Miao,^d,^‡ Mingwei Yu,^a^ Xixi Wu,^a^ Yuanhang Liu,^a^ Jie Fu,^a^ Junwei Chen,^a^ Liya Ma,^e^ Xiangru Chen,^a^ Ximing Peng,^a^ You Zhen,^b,^* Chuchao Zhou^a,^*

a Department of Plastic Surgery, Tongren Hospital of Wuhan University (Wuhan Third Hospital), Wuhan, 430060, China

b Division of Biliary Surgery, Department of General Surgery, West China Hospital, Sichuan University, Chengdu, 610041, China

c Department of Plastic Surgery, Beijing Hospital of integrated traditional Chinese and Western Medicine, Beijing, 100038, China

d Department of Dermatology, Renmin Hospital of Wuhan University, Wuhan, 430060, China

e The Centre of Analysis and Measurement of Wuhan University, Wuhan University, Wuhan, 430072, PR China

*** Corresponding authors**

[youzhen@wchscu.cn](mailto:benjamin.wzx@163.comyouzhen@wchscu.cn) (You Zhen)

[chuchaozhou@163.com](mailto:chuchaozhou@163.com) (Chuchao Zhou)

**
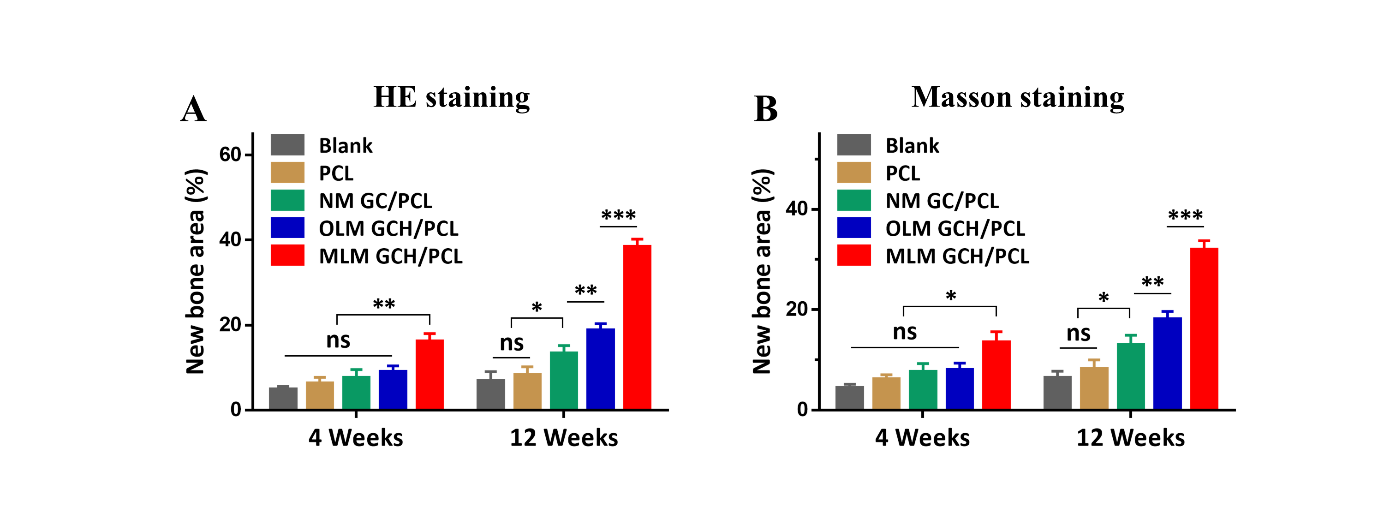
**

**Fig. S1.** Quantitative analysis of new bone area in HE and Masson staining. * p<0.05, ** p<0.01, *** p<0.001, and ns: no significance.

**
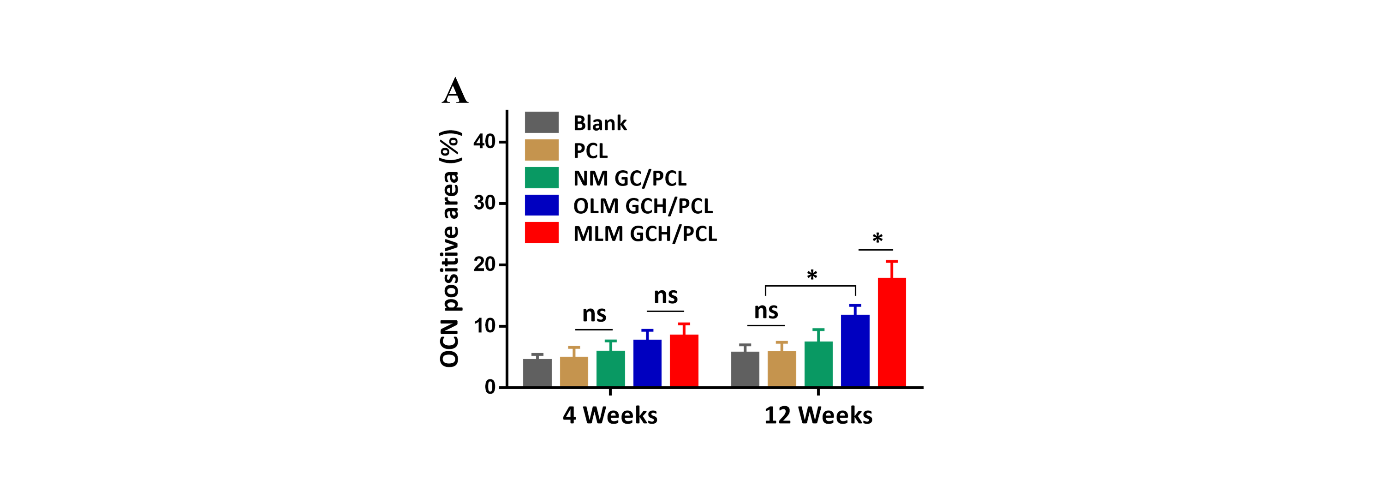
**

**Fig. S2.** Quantitative analysis of the expression level of OCN. * p<0.05, and ns: no significance.


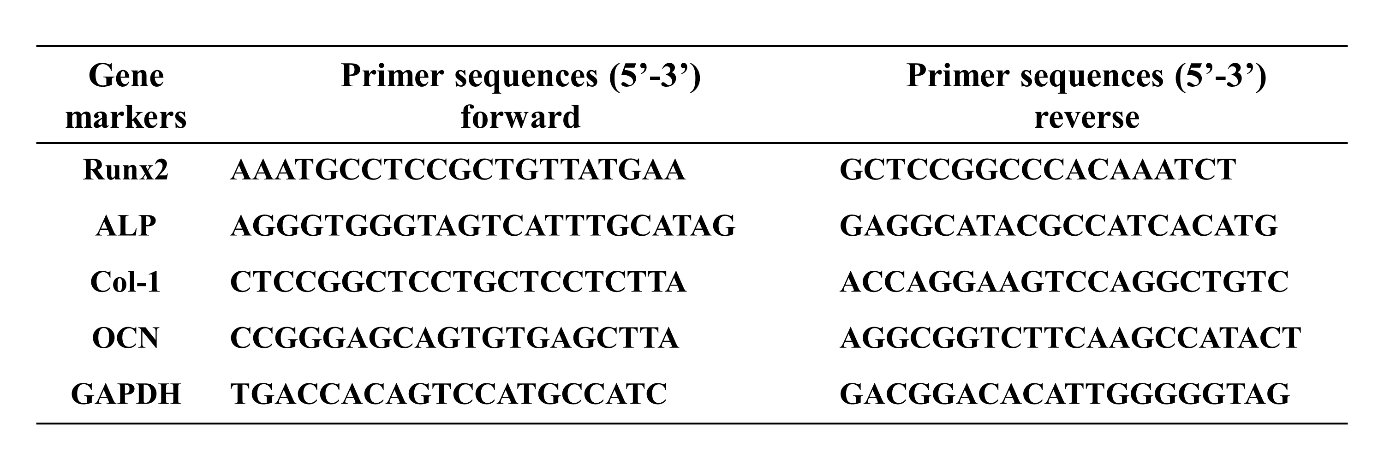


**Table. S1.** Primer sequences used for gene expression analysis by qRT-PCR. Runx2: runt-related transcription factor 2, ALP: alkaline phosphatase, Col-1: collagen type-1, OCN: osteocalcin, GAPDH: glyceraldehyde 3-phosphate dehydrogenase.
